# Nutritional status and household food security of pregnant women: insights from the Nutritional Status of Expectant Mothers and their Newborn Infants (NuEMI) study in South Africa

**DOI:** 10.1017/S1368980025100815

**Published:** 2025-08-11

**Authors:** Janet Adede Carboo, Jennifer Ngounda, Liska Robb, Marizeth Jordaan, Corinna May Walsh

**Affiliations:** Department of Nutrition and Dietetics, School of Health and Rehabilitation Sciences, Faculty of Health Sciences, University of the Free State, Bloemfontein, South Africa

**Keywords:** Nutrient intake, Pregnancy, Household food security, Estimated average requirement, Recommended dietary allowance

## Abstract

**Objective:**

To describe the nutritional intake and status of pregnant women in Bloemfontein and compare across different household food security categories.

**Design:**

Cross-sectional.

**Setting:**

Pelonomi Tertiary Hospital.

**Participants:**

427 pregnant women were interviewed using a standard questionnaire and a quantitative FFQ to collect socio-demographic, HIV status, household food security, supplement and dietary intake data. Weight and height were measured using standard anthropometric techniques and capillary blood taken by finger-prick for anaemia, Fe and inflammation status assessment.

**Results:**

26·7 % of participants were food secure, while 11·5 %, 32·1 % and 29·5 % experienced mild, moderate and severe food insecurity, respectively. 54·5 %, 41·7 % and 31·1 % were obese, anaemic and Fe deficient. Median energy intake was 8808 (6978–9223) KJ/d, with no significant differences between the food security groups (*P* = 0·517). Based on the dietary reference intakes, 98·1 % met the estimated average requirement (EAR) for carbohydrates, but the majority had sub-optimal intake of protein (58·3 %), fibre (60·9 %), pantothenic acid (67·0 %), vitamins C (65·6 %), D (68·4 %), E (59·0 %) and K (61·8 %), potassium (99·8 %), dietary Ca (95·8 %) and Fe (80·8 %). Compared with the moderately and severely food-insecure counterparts, food-secure participants had a higher intake of animal protein (*P* < 0·001), total fat (*P* = 0·014), monounsaturated fat (*P* = 0·002), vitamins B_12_ (*P* = 0·014), C (*P* < 0·001) and D (*P* = 0·003) and dietary Ca (*P* = 0·001). Dietary folate intake was below the EAR in 69·9 %, but was higher among severely food-insecure participants (463·94 (327·39, 609·71) µg than food secure (378·49 (265·99, 496·15) µg, *P* = 0·007)).

**Conclusion:**

The findings show widespread inadequate nutrient intake among pregnant women in Bloemfontein, with food-insecure women showing significantly lower intake of specific nutrients.

Adequate nutrient intake during pregnancy is essential for supporting both maternal health and optimal fetal development. In contrast, poor nutritional intake during this period is a significant risk factor for short- and long-term maternal and fetal complications such as intrauterine growth restriction, low birth weight, neonatal infection, still birth, maternal undernutrition, anaemia and preeclampsia among many others^(1)^. The adequacy of an individual’s nutritional intake is often impacted by their level of food security, which is defined as the consistent accessibility to sufficient, safe and nutritious food for normal growth and development and a healthy life^(2)^. However, in Africa, food insecurity is widespread, affecting approximately 868 million people, with pregnant women being disproportionately vulnerable^(3)^. Pregnant women living in food-insecure households are at a higher risk of inadequate nutritional intake and micronutrient deficiencies, which can negatively affect birth outcomes^(4)^. In developing countries including South Africa, the nutritional intake of pregnant women is influenced by several factors such as socio-economic status, educational level, marital status, family size and ethnicity^(5)^. In urban Johannesburg, South Africa, pregnant women were observed to have borderline poor-quality diet, high in total fat, saturated fat and Na, with 63 % of them consuming less than half of the recommended amount of vegetables per day^(6)^. Additionally, maternal micronutrient deficiencies, including Fe, vitamin A, folate and Zn, are also widespread in sub-Saharan Africa, including South Africa^(7)^. Adequate intake of these nutrients is crucial for preventing congenital anomalies, supporting immune function and promoting healthy fetal growth and development^(8)^. Despite the recognised importance of these nutrients, many pregnant women in sub-Saharan Africa do not have an adequate nutrient intake, partly due to limited access to diverse and nutrient-rich foods at the household level^(9)^. In South Africa, approximately 21 % of households (3·72 million) experience food insecurity^(10)^. However, there is limited comprehensive data on how nutrient intake and status of pregnant women differ according to household food security status in South Africa. Assessing this association is crucial for developing relevant interventions and policies that support maternal nutrition and fetal health in South Africa. Hence, this study aimed to provide a comprehensive overview of the nutritional intake of pregnant women in the Free State Province of South Africa and examine how these intakes vary based on household food security.

## Methodology

### Study design, site and participants

This cross-sectional study was nested in the Nutritional Status of Expectant Mothers and their Newborn Infants (NuEMI) study, conducted at Pelonomi Tertiary Hospital in Bloemfontein, South Africa, from May 2018 to April 2019. Pelonomi Hospital is a referral centre for high-risk pregnancies in the Mangaung district, to which pregnant women with conditions such as obesity, hypertension, diabetes mellitus, adolescent age (<18 years), advanced age (>35 years), two or more previous cesarean sections, multiple pregnancies and previous poor pregnancy outcomes (neonatal death and preterm delivery) are referred. All pregnant women attending their routine antenatal care visits at the hospital were invited to participate in the study after being informed about the study by trained fieldworkers. Using a convenience sampling approach, all interested pregnant women were consecutively recruited for eligibility screening and participation. Eligible participants were required to be at least 18 years old and able to understand and speak English, Afrikaans or SeSotho. Those with multiple pregnancies (i.e. pregnant with two or more babies) were not included in this sub-study because of their increased risk of complications compared with those with singleton pregnancies^(11)^. Eligible pregnant women, regardless of their stage of pregnancy, were enrolled after providing written informed consent.

### Data collection

#### Socio-demographic, health and anthropometric information

In a structured interview by trained fieldworkers, information including age, level of education, marital status, employment status and household income including social grants were collected from participants using a questionnaire adapted from the ‘Assuring Health in the Free State’ study^(12)^. Additionally, health-related data, such as smoking or tobacco use, previous pregnancies, HIV status, supplement and medication use, were collected. Participants’ weight was measured using a high-capacity digital scale (Seca 876; Germany), which was calibrated daily and recorded to the nearest 0·1 kg. Participants removed their shoes, emptied their pockets and wore minimal clothing before the weight measurement. Height was measured with a standard stadiometer (Seca 213) and recorded to the nearest 0·1 cm. The weight and height measurements were taken in accordance with standard anthropometric techniques established by the International Society for the Advancement of Kinanthropometry^(13)^. Gestational body mass index was calculated using an algorithm that adjusts BMI by gestational age^(14)^.

#### Dietary intake

A 329-item quantitative FFQ (QFFQ) was used to assess participants’ dietary intake, focusing on food intake within the previous 28 d. Prior to its use in this study, the QFFQ had been validated for the population in the Women’s Health Study in the Free State, South Africa^(15)^ and has shown to be reproducible in similar adult populations in South Africa^(16)^. This QFFQ has also been previously used among pregnant women in the Nutrition during Pregnancy and Early Development study in Johannesburg, South Africa, in which minor amendments were made due to vernacular differences in certain terminologies^(17)^. Trained field workers administered the QFFQ in structured interviews in the preferred languages of the participants. Additionally, participants had to indicate if they were taking any supplements, where they obtained them and the frequency of their intake. The total nutrient intake of participants including both dietary and supplement intake was estimated at the South African Medical Research Council using the most recent South African Food Composition Database, which considers the nutrient content of fortified foods. The adequacy of the nutrient intakes was determined by comparing the nutrient intakes with the dietary reference intakes of the Institute of Medicine^(18)^, including the recommended dietary allowance (RDA), estimated average requirement (EAR), adequate intake (AI) and tolerable upper limit (UL).

#### Household food security

The Household Food Insecurity Access Scale, consisting of nine questions, assessed whether household members had experienced concerns about insufficient food, altered their diets, reduced meal sizes or frequency or gone to bed hungry due to limited household food availability in the last 4 weeks^(19)^. A response of ‘yes’ to each of the nine questions was followed up by a question to ascertain the frequency of occurrence. The frequency of occurrence within the previous 4 weeks was classified as rarely (once or twice), sometimes (three to ten times) or often (more than ten times) and scored accordingly. Participants were grouped according to the following categories based on their scores: food secure, mildly food insecure, moderately food insecure and severely food insecure^(19)^.

#### Anaemia, iron and inflammatory status

Capillary blood from a finger-prick was used to assess concentrations of Hb, ferritin, soluble transferrin receptor, C-reactive protein and alpha-1-acid glycoprotein of the participants. Anaemia was defined as Hb <11 g/dl based on the WHO Hb cut-off for pregnancy^(20)^. Fe status was categorised as (1) Fe deficiency (ID) (i.e. adjusted ferritin <15 µg/l)^(21)^; (2) Fe-deficiency erythropoiesis (i.e. soluble transferrin receptor > 8·3 µg mg/l)^(22)^; and (3) Fe deficiency anaemia ((i.e. ferritin <15 µg/l and Hb <11 g/dl)^(20)^. C-reactive protein > 5 mg/l or alpha-1-acid glycoprotein > 1 g/l was defined as elevated inflammation. Further details on the assessment of Fe and inflammation status in this study are described elsewhere^(23)^.

### Statistical analysis

Statistical analyses were performed using Statistical Package for Social Sciences software, Version 27. The distribution of the data was tested using Kolmogorov–Smirnov test, histograms and Q-Q plots. Using descriptive statistics, the continuous characteristics of the participants were described using medians with 25th and 75th percentiles (Q1, Q3). Frequencies and percentages were used to describe categorical characteristics of participants. Differences in continuous variables between the groups were assessed using the Kruskal–Wallis test. Categorical variables were compared using *χ* ^2^ tests. *P* values of <0·05 were considered significant.

## Results

### Socio-demographic and health characteristics of participants

Table 1 shows the characteristics of the study participants stratified by household food security status. Four hundred and twenty-seven participants with a median (Q1, Q3) age of 32 (27, 37) years were enrolled into this sub-study and included in the analysis. At enrollment, the median (Q1, Q3) gestational age of the participants was 32 (26, 36) weeks. Majority of the participants (72·6 %) were in their third trimester, while 114 (26·7 %) and 3 (0·7 %) were in their second and first trimesters, respectively. Forty-three (10 %) of the participants were primigravid. The majority (56·7 %) of the women were in a relationship but unmarried, while 36·0 % were married. The median (Q1, Q3) gestational body mass index of the participants was 31·3 (24·6, 37·7) kg/m^2^, with majority (54·5 %) being obese (gestational body mass index ≥ 29 kg/m^2^), while 6·5 % were underweight (gestational body mass index ≥ 10 to < 19·8 kg/m^2^). Most participants experienced mild (11·5 %), moderate (32·1 %) to severe (29·5 %) household food insecurity, while only 26·7 % were food secure. A higher proportion of underweight (39·3 %), normal weight (35·8 %) and overweight (37·0 %) women were severely food insecure, compared with 23·5 % of the obese (*P* < 0·001). About 31 % of the participants were HIV positive and on anti-retroviral treatment. Moreover, a significantly higher proportion of participants living with HIV infection (74·2 %) were moderately and severely food insecure compared with those without HIV infection (55·9 %) (*P* = 0·002).

**Table 1.** Socio-demographic and health characteristics of participants according to household food security classification

	Total group (*n* 427)		Food secure (*n* 114)		Mildly food insecure (*n* 49)		Moderately food insecure (*n* 137)		Severely food insecure (*n* 126)		
Characteristics	Median	Q1, Q3	Median	Q1, Q3	Median	Q1, Q3	Median	Q1, Q3	Median	Q1, Q3	*P* value
Age (years)	32	27, 37	31	26, 36	34	27, 37	31	27, 36	32	27, 36	0·564^*^
Gestational BMI (kg/m^2^)	31	25, 38	34	25, 39	32	25, 39	31	26, 38	28	23, 36	0·072^*^
	*n*	%	*n*	%	*n*	%	*n*	%	*n*	%	
Gestational BMI categories											
Underweight (*GBMI ≥ 10 to < 19·8 kg/m^2^)*	28	6·5	6	5·3	1	2·0	10	7·3	11	8·7	**<0·001**
Normal weight *(GBMI ≥ 19·8 to < 26·1 kg/m^2^)*	106	24·8	26	22·8	13	26·5	28	20·4	38	30·2	
Overweight *(GBMI ≥ 26·1 to < 29 kg/m^2^)*	46	10·7	9	7·9	4	8·2	16	11·7	17	13·5	
Obese *(GBMI ≥ 29 kg/m^2^)*	242	54·5	72	63·2	31	63·3	82	59·9	57	45·2	
Highest level of education											
Primary school	31	7·3	5	4·4	4	8·2	4	2·9	18	14·3	**0·003**
Grade 8–10	111	26·0	18	15·8	10	20·4	39	28·5	44	34·9	
Grade 11–12	238	55·7	74	64·9	30	61·2	78	56·9	55	43·7	
Tertiary	46	10·8	17	14·9	5	10·2	16	11·7	8	6·3	
Don’t know	1	0·2	0	0·0	0	0·0	0	0·0	1	0·8	
Marital status											
Married	155	36·0	41	36·0	16	32·7	55	40·1	42	33·3	0·329
Not married, but in a relationship	242	56·7	65	57·0	30	61·2	77	56·2	70	55·6	
Not married, and not in a relationship	21	4·9	5	4·4	1	2·0	4	2·9	11	8·7	
Divorced/Separated	4	0·9	0	0·0	1	2·0	0	0·0	3	2·4	
Widowed	2	0·5	2	1·8	0	0·0	0	0·0	0	0·0	
Other	3	0·7	1	0·9	1	2·0	1	0·7	0	0·0	
Tobacco Use											
History of Smoking pre-pregnancy	83	19·4	16	14·0	7	14·3	28	20·4	32	25·4	0·189
Smoking during pregnancy	30	7·0	3	2·6	2	4·1	10	7·3	15	11·9	0·328
History of Snuffing tobacco prior to pregnancy	90	21·2	14	12·3	10	20·4	28	20·7	38	30·4	**0·017**
Snuffed tobacco during pregnancy	33	7·7	6	46·2	5	10·2	8	5·8	14	11·1	0·565
Alcohol Use											***P*****-value^†^**
History of drinking alcohol pre-pregnancy	309	72·4	82	71·9	39	79·6	94	68·6	94	74·6	0·268
Alcohol use during pregnancy	41	9·6	6	5·3	8	16·3	8	5·8	19	15·1	
Monthly household income (ZAR)											
None	1	0·2	0	0·0	0	0·0	0	0·0	1	0·8	**<0·001**
100–500	22	5·2	0	0·0	1	2·0	4	2·9	17	13·5	
501–1000	34	8·0	3	2·7	3	6·1	7	5·1	21	16·7	
1001–3000	126	29·6	24	21·2	15	30·6	43	31·6	44	34·9	
3001–5000	95	22·4	26	23·0	8	16·3	38	27·9	23	18·3	
>5000	127	29·9	55	48·7	17	34·7	39	28·7	16	12·7	
Don’t know	20	4·7	5	4·4	5	10·2	5	3·7	4	3·2	
Employment status											
Full-time employed	89	20·8	33	28·9	7	14·3	29	21·2	20	15·9	0·097
Part-time employed	49	11·5	14	12·3	5	10·2	15	10·9	15	11·9	
Unemployed	222	52·0	51	44·7	25	51·0	72	52·6	74	58·7	
Self-employed	34	8·0	7	6·1	8	16·3	9	6·6	9	7·1	
Housewife by choice	22	5·2	5	4·4	4	8·2	8	5·8	5	4·0	
Other	11	2·6	4	3·5	0	0·0	4	2·9	3	2·4	
Access to running water											
Indoor water	216	50·6	78	68·4	26	53·1	63	46·0	49	38·9	**<0·001**
Own tap outside the house	127	29·7	25	21·9	12	24·5	49	35·8	41	32·5	
Share tap with other households	84	19·7	11	9·6	11	22·4	25	18·2	36	28·6	
Access to toilet facilities											
Flush toilet inside the house	128	30·0	46	40·6	13	26·5	41	29·9	28	22·2	**0·046**
Own flush toilet outside the house	147	34·4	35	30·7	19	38·8	48	35·0	45	35·7	
Share an outside toilet with other households	49	11·5	11	9·6	8	16·3	16	11·7	14	11·1	
Use of bucket system	39	9·1	5	4·4	4	8·2	11	8·0	18	14·3	
Own pit toilet outside the house	64	15·0	17	14·9	5	10·2	21	15·3	21	16·7	
HIV status											
Positive	132	30·9	20	17·5	13	26·5	46	33·6	52	41·3	**0·002**
Negative	295	69·1	94	82·5	36	73·5	91	66·4	74	58·7	
Anaemia status											
Anaemic *(Hb < 11 g/dl)*	178	41·7	41	36·0	16	32·7	59	43·1	62	49·2	0·139
Non-anemic *(Hb ≥ 11 g/dl)*	249	58·3	73	64·0	33	67·3	78	56·9	64	50·8	
Iron status											
ID *(ferritin <15 µg/l)*	133	31·1	39	34·2	17	34·7	36	26·3	41	32·5	0·580
Non-ID *(ferritin ≥15 µg/l)*	294	68·9	75	65·8	32	65·3	101	73·7	85	67·5	
IDA *(ferritin <15 µg/l and Hb <11 g/dl)*	81	18·9	21	18·4	10	20·4	22	16·1	28	22·2	0·702
Non-IDA	346	81·1	93	81·6	39	79·6	115	83·9	98	77·8	
IDE *(sTfR > 8·3µg mg/l)*	42	9·8	6	5·3	7	14·3	14	10·2	15	11·9	0·340
Non-IDE *(sTfR ≤ 8·3µg mg/l)*	385	90	108	94·7	42	85·7	123	89·8	111	88·1	
Inflammation status											
Elevated CRP *(> 5 mg/l)*	293	68·6	79	69·3	38	77·6	95	69·3	81	64·3	0·271
Normal CRP *(≤ 5 mg/l)*	134	31·4	35	30·7	11	22·4	42	30·7	45	35·7	
Elevated AGP *(>1 g/l)*	10	2·3	4	3·5	0	0·0	3	2·2	3	2·4	0·756
Normal AGP *(≤ 1 g/l)*	417	97·7	110	96·5	49	100·0	134	97·8	123	97·6	

GBMI, gestational body mass index; ZAR, South African rand; ID, Fe deficiency; IDA, Fe deficiency anaemia; IDE, Fe deficiency erythropoiesis; CRP, C-reactive protein; AGP, alpha-1-acid glycoprotein.

* Kruskal–Wallis test.

† Pearson *χ* ^2^ test.

Statistical significance (*P* < 0.05).

More than half (55·7 %) of the participants completed grade 12, while only 10·8 % had tertiary education. A greater proportion of participants who had achieved lower levels of education (i.e. primary school (58·1 %) and grade 8–10 (39·6 %)) were severely food insecure compared with those who had higher education (i.e. 23·1 % in those who completed grade 11–12 and 17·4 % in those with tertiary education (*P* = 0·003). About 52 % of the participants were unemployed, and 43 % had a monthly household income of R3000 (approximately $168) or less. A significant majority of participants in the lowest monthly income categories (i.e. 77 % in the R100–500 (≈ $6–28) and 62 % in the R501–1000 (≈ $28·1–56)) were severely food insecure (*P* < 0·001).

Approximately 19 % and 21 % of the participants had a history of smoking and tobacco snuffing, respectively, prior to pregnancy, and among the latter, a significant majority (73 %) were moderately and severely food insecure. Moreover, 30 (7 %) and 33 (8 %) continued smoking and snuffing tobacco during pregnancy, respectively. There were no significant differences in the proportions of participants with or without anaemia, ID, Fe deficiency anaemia and Fe-deficiency erythropoiesis and elevated inflammation across the different household food security categories.

### Energy and macronutrient intake

Table 2 illustrates the daily energy and macronutrients intake of participants according to their household food security status. The median (Q1, Q3) daily energy intake was 8808 (6978, 9223) KJ, with no significant differences observed across the different food security statuses (*P* = 0·517). Majority (98·1 %) of the participants had a median daily carbohydrate intake above the EAR of 135 g/d for pregnant women^(18)^. The median (Q1, Q3) daily protein intake was 64·81 g (50·18, 85·26 g), with 58·2 % of the participants consuming less than the RDA of 71 g/d. Among them, a significantly higher proportion (77 %) were from food-insecure households (*P* = 0·038). The food-secure participants had a significantly higher median protein intake of 71·35 g (52·27, 92·75 g) compared with 63·18 g (48·54, 83·08 g) in the food-insecure group (*P* = 0·030). Additionally, animal protein intake was significantly higher among the food-secure participants (i.e. 31·32 (19·18, 45·35) g) compared with those from severely food-insecure households (24·02 (14·57–40·49) g; *P* < 0·001). Compared with the severely food-insecure participants, the food-secure participants had a significantly higher daily intake of total fats (i.e. 58·70 (44·86, 83·16) g *v*. 73·69 (51·15, 99·28) g; *P* = 0·004), saturated fats (i.e. 14·32 (8·78, 23·06) g *v*. 22·01 (15·19, 29·33) g; *P* < 0·001), monounsaturated fat (i.e. 15·80 (8·88, 26·50) g *v*. 21·99 (16·66, 32·04) g; *P* < 0·001) and cholesterol (151·27 (83·59, 281·24) g *v*. 248·04 (145·50, 406·28) g; *P* = 0·001). Approximately 61 % of all the participants did not meet the AI for fibre.

**Table 2.** Dietary energy, macronutrient and fiber intake of participants by household food security classification

	Total (*n* 427)		Food secure (*n* 114)		Mildly food insecure (*n* 49)		Moderately food insecure (*n* 137)		Severely food insecure (*n* 126)		
Nutrient per day	Median	Q1, Q3	Median	Q1, Q3	Median	Q1, Q3	Median	Q1, Q3	Median	Q1, Q3	*P* value^*^
Energy (KJ)	8808	6978, 9223	9533	7412, 11 330	8268	7080, 10 130	8707	6765, 10 934	8374	6411, 10 992	0·517
Macronutrients											
Carbohydrate (g)	321·10	254·01, 393·20	327·72	253·54, 382·96	296·88	254·91, 374·25	312·28	257·57, 390·03	331·94	249·86, 431·30	0·841
Dietary fibre (g)	25·19	19·89, 31·99	24·42	18·46, 31·26	23·93	19·06, 29·67	25·72	20·63, 30·80	25·24	20·11, 33·86	0·542
Total protein (g)	64·81	50·18, 85·26	71·35	52·27, 92·75	65·05	52·07, 82·23	64·47	51·96, 84·37	58·70	44·86, 83·16	0·151
Plant protein (g)	31·94	24·65, 40·03	31·78	23·55, 38·09	29·90	23·11, 38·64	31·08	25·75, 41·17	32·57	24·06, 43·01	0·506
Animal protein (g)	31·32	19·18, 45·35	35·89^a,b^	25·89, 52·22	32·89	24·15, 48·26	30·06^b^	18·43, 42·40	24·02^a^	14·57, 40·49	**<0·001**
Total fat (g)	63·19	42·06, 90·46	73·69^c^	51·15, 99·28	60·07	47·54, 80·95	61·47	41·48, 88·14	53·71^c^	36·17, 87·70	**0·014**
Saturated fat (g)	17·31	11·23, 25·21	22·01^d,e^	15·19, 29·33	17·18	13·73, 24·35	16·87^e^	11·45, 25·15	14·32^d^	8·78, 23·06	**<0·001**
Monounsaturated fat (g)	18·96	11·92, 28·79	21·99^f^	16·66, 32·04	18·96	12·20, 26·42	18·61	12·68, 27·88	15·80^f^	8·88, 26·50	**0·002**
Polyunsaturated fat (g)	17·21	11·30, 25·42	19·52	13·35, 26·15	15·59	12·74, 23·42	18·54	11·31, 25·91	15·26	9·11, 24·50	0·131
Total trans fats (g)	0·67	0·31, 1·30	0·85	0·42, 1·46	0·72	0·29, 1·20	0·65	0·32, 1·30	0·63	0·24, 1·23	0·151
Cholesterol (mg)	193·07	117·03, 341·70	248·04^g^	145·50, 406·28	193·07	141·81, 282·05	202·82	111·99, 341·81	151·27^g^	83·59, 281·24	**0·004**
Nutrient intake compared with dietary reference intake											
	*n*	%	*n*	%	*n*	%	*n*	%	*n*	%	*P*-value^†^
Carbohydrate											
Above the EAR (≥ 135 g/d)	419	98·1	112	98·2	49	100	135	98·5	122	96·8	0·690
Below the EAR (<135 g/d)	8	1·9	2	1·8	0	0·0	2	1·5	4	3·2	
Total protein											
Above the RDA (≥ 71 g/d)	178	41·7	57	50·0	17	34·7	54	39·4	50	39·7	0·245
Below the RDA (< 71 g/d)	249	58·3	57	50·0	32	65·3	83	60·6	76	60·3	
Dietary fiber											
Above the AI (≥28 g)	167	39·1	40	35·1	18	36·7	50	36·5	58	46·0	0·251
Below the AI (< 28 g)	260	60·9	74	64·9	31	63·3	87	63·5	68	54·0	

EAR, estimated average requirement; RDA, recommended dietary allowance; AI, adequate intake.

* Kruskal–Wallis test.

† Pearson *χ* ^2^ test.

Post hoc test using Bonferroni correction (a: *P* < 0.001; b: *P* = 0.064; c: *P* = 0.004; d: *P* < 0.001; e: *P* = 0.028; f: *P* < 0.001; g: *P* = 0.001).

Statistical significance (*P* < 0.05).

### Dietary and supplemental intake of vitamins

**Figure 1.**
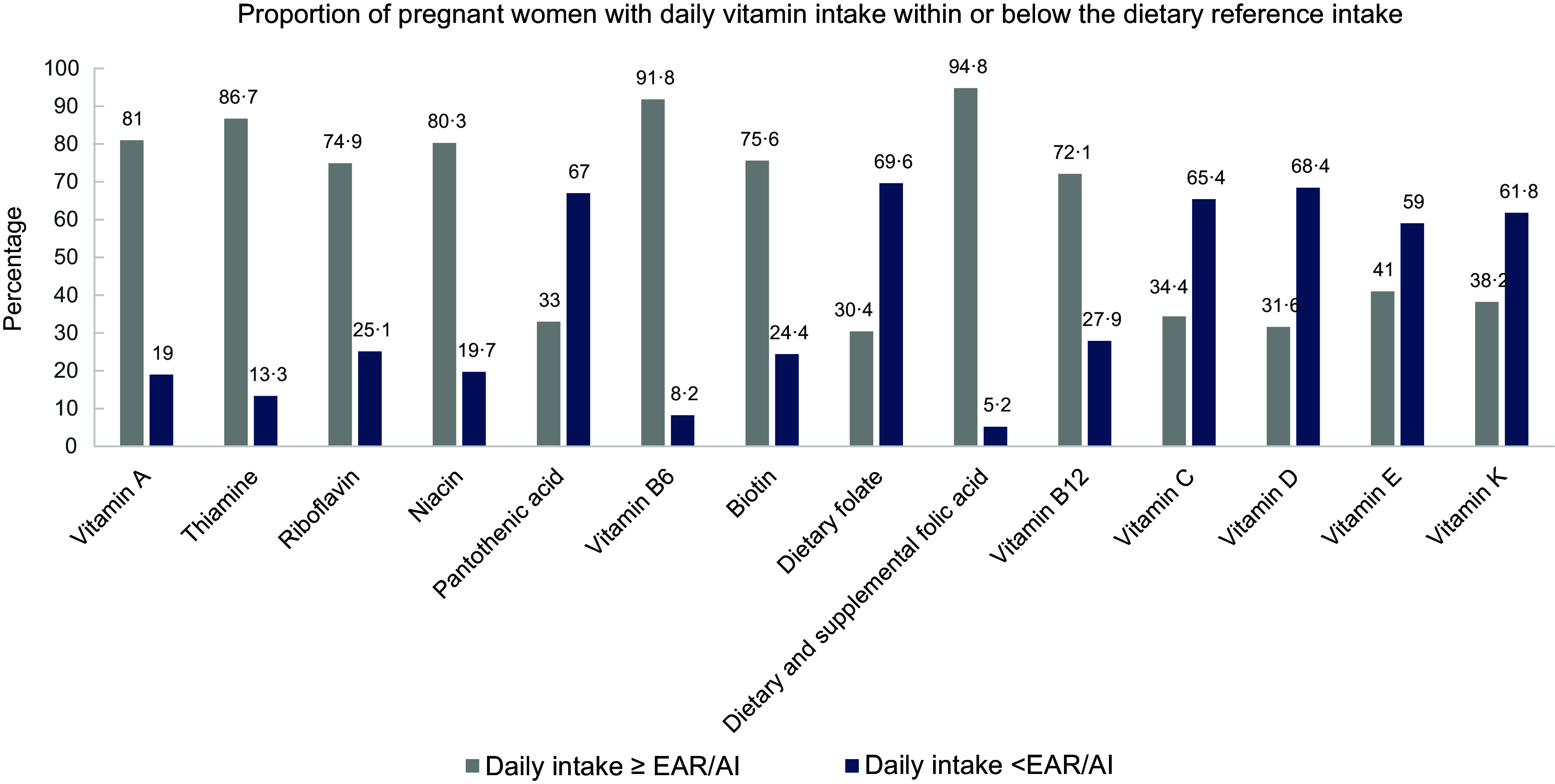
Proportion of pregnant women with dietary and supplemental vitamin intakes above or below daily requirements.

Table 3 presents the intake of vitamins from both dietary and supplemental sources stratified by household food security status. Figure 1 also shows the proportion of pregnant women with daily vitamin intake within or below the dietary reference intakes. The median (Q1, Q3) dietary intake of folate was 412·12 (309·14, 547·75) µg, with the severely food-insecure participants having a significantly higher daily intake (463·94 (327·39, 609·71) µg) compared with their food-secure counterparts (378·49 (265·99, 496·15) µg, *P* = 0·007). Only 30·4 % of participants met the EAR of 520 µg for folate through dietary intake. However, with the addition of 5 mg prenatal folic acid supplements provided by the hospital, 94·8 % of the pregnant women achieved an intake far above the recommended intake. Furthermore, the median (Q1, Q3) daily intake of vitamin B_12_ in the total group was 3·69 µg (2·03, 6·57 µg), and about 72·1 % of the participants had intakes above the EAR of 2·2 µg. However, the intake varied significantly across the different household food security groups (*P* = 0·010), with participants from severely food-insecure households having a significantly lower intake (2·97 µg (1·60, 6·09 µg)) compared with those from food-secure households (4·34 µg (2·91, 8·16 µg), *P* = 0·001).

**Table 3.** Dietary and supplemental intake of vitamins among the pregnant women

	Total (*n* 427)		Food secure (*n* 114)		Mildly food insecure (*n* 49)		Moderately food insecure (*n* 137)		Severely food insecure (*n* 126)		
Nutrient per day	Median	Q1, Q3	Median	Q1, Q3	Median	Q1, Q3	Median	Q1, Q3	Median	Q1, Q3	*P* value^*^
Vitamin A (µgRAE)	1007	638, 1786	1112	714, 2034	1006	647, 1940	952·11·	631·28, 1633	1007	561, 1748	0·362
Thiamin (mg) (B1)	1·94	1·46, 2·44	1·88	1·37, 2·36	1·79	1·41, 2·27	1·94	1·59, 2·42	1·99	1·47, 2·74	0·299
Riboflavin (mg) (B2)	1·68	1·20, 2·33	1·83	1·29, 2·42	1·73	1·17, 2·17	1·70	1·20, 2·33	1·55	1·08, 2·30	0·240
Niacin (mg) (B3)	20·93	15·51, 28·52	22·63	17·23, 28·93	20·13	15·26, 28·13	21·40	16·38, 28·61	19·44	13·52, 28·15	0·172
Pantothenic acid (mg) (B5)	4·98	3·48, 6·68	5·25	3·89, 7·06	5·02	3·91, 6·87	5·10	3·45, 6·64	4·60	2·99, 6·42	0·166
Vitamin B_6_ (mg)	3·32	2·44, 4·52	3·35	2·40, 4·33	3·18	2·50, 4·21	3·49	2·57, 4·77	3·21	2·22, 4·55	0·490
Biotin (µg)	40·56	30·11, 54·55	41·31	29·37, 56·82	39·98	31·37, 51·85	40·07	31·13, 53·96	40·51	28·87, 57·61	0·997
Dietary folate (µg)	412·12	309·14, 547·75	378·49^a^	265·99, 496·15	393·06	290·41, 579·98	403·35	324·74, 543·03	463·94^a^	327·39, 609·71	**0·021**
Dietary folate with supplements (µg)	5393	5274, 5532	5342^a,b^	5217, 5481	5392	5254, 5575	5396^a^	5311, 5705	5428^b^	5300, 5584	**0·019**
Vitamin B_12_ (µg)	3·69	2·03, 6·57	4·34^c^	2·91, 8·16	3·84	2·41, 7·05	3·61	2·16, 5·78	2·97^c^	1·60, 6·09	**0·014**
Vitamin C (mg)	51·44	27·74, 87·07	71·96^d,e,f^	38·94, 118·18	42·40^d^	25·39, 62·77	40·88^e^	24·83, 86·05	44·62^f^	22·85, 75·44	**<0·001**
Vitamin D (µg)	3·61	1·67, 5·53	4·49^g^	2·49, 6·13	3·55	1·82, 5·16	3·74	1·73, 5·99	2·49^g^	1·16, 4·87	**0·003**
Vitamin E (mg)	10·55	7·40, 15·97	11·64	8·38, 16·26	10·28	8·32, 15·09	10·63	7·66, 16·83	9·45	6·30, 15·08	0·190
Vitamin K (µg)	34·99	28·24, 119·28	66·33	34·25, 124·88	57·75	34·08, 93·74	65·51	26·78, 120·90	60·62	22·90, 115·07	0·350
Nutrient intake compared with dietary reference intake											
	*n*	%	*n*	%	*n*	%	*n*	%	*n*	%	*P*-value^†^
Vitamin A											
Above the EAR (≥ 550 µg)	346	81·0	99	86·8	41	83·7	109	79·6	96	76·2	0·280
Below the EAR (< 550 µg)	81	19·0	15·	13·2	8·	16·3	28·	20·4	30·	23·8	
Above UL (> 3000 µg)	30	7·0	12	10·5	4	8·2	6	4·4	8	6·3	0·424
Below the UL (≤ 3000 µg)	397	93·0	102	89·5	45	91·8	131	95·6	118	93·7	
Thiamine											
Above the EAR (≥ 1·2 mg)	370	86·7	94	82·5	43	87·8	126	92·0	106	84·1	0·200
Below the EAR (< 1·2 mg)	57	13·3	20	17·5	6	12·2	11	8·0	20	15·9	
Riboflavin											
Above the EAR (≥ 1·2 mg)	320	74·9	92	80·7	35	71·4	103	75·2	89	70·6	**0·417**
Below the EAR (< 1·2 mg)	107	25·1	22	19·3	14	28·6	34	24·8	37	29·4	
Niacin											
Above the EAR (≥ 14 mg)	343	80·3	95	83·3	40	81·6	115	83·9	92	73·0	0·175
Below the EAR (< 14 mg)	84	19·7	19	16·7	9	18·4	22	16·1	34	27·0	
Above UL (> 35 mg)	36	8·4	12	10·5	3	6·1	11	8·0	10	7·9	0·887
Below the UL (≤ 35 mg)	391	91·6	102	89·5	46	93·9	126	92·0	116	92·1	
Pantothenic acid											
Above the AI (≥ 6 mg)	141	33·0	46	40·4	15	30·6	43	31·4	37	29·4	0·365
Below the AI (< 6 mg)	286	67·0	68	59·6	34	69·4	94	68·6	89	70·6	
Vitamin B_6_											
Above the EAR (≥ 1·6 mg)	392	91·8	103	90·4	45	91·8	128	93·4	115	91·3	0·919
Below the EAR (< 1·6 mg)	35	8·2	11	9·6	4	8·2	9	6·6	11	8·7	
Above UL (> 100 mg)	0	0·0	0	0·0	0	0·0	0	0·0	0	0·0	**–**
Below the UL (≤ 100 mg)	427	100·0	114	100·0	49	100·0	137	100·0	126	100·0	
Biotin											
Above the AI (≥ 30 mg)	323	75·6	83	72·8	40	81·6	107	78·1	92	73·0	0·610
Below the AI (< 30 mg)	104	24·4	31	27·2	9	18·4	30	21·9	34	27·0	
Dietary folate											
Above the EAR (≥ 520 mg)	130	30·4	23	20·2	19	38·8	40	29·2	48	38·1	0·023
Below the EAR (< 520 mg)	297	69·6	91	79·8	30	61·2	97	70·8	78	61·9	
Dietary and supplemental folic acid											
Above the EAR (≥ 520 mg)	404	94·8	105	92·1	46	93·9	131	95·6	121	96·8	0·551
Below the EAR (< 520 mg)	22	5·2	9	7·9	3	6·1	6	4·4	4	3·2	
Above UL (> 1000 mg)	394	92·5	101	25·6	45	11·4	130	33·0	117	39·7	0·412
Below the UL (≤ 1000 mg)	32	7·5	13	11·4	4	8·2	7	5·1	8	6·4	
Vitamin B_12_											
Above the EAR (≥ 2·2 µg)	308	72·1	91	79·8	38	77·6	101	73·7	78	61·9	**0·010**
Below the EAR (< 2·2 µg)	119	27·9	23	20·2	11	22·4	36	26·3	48	38·1	
Vitamin C											
Above the EAR (≥ 70 mg)	147	34·4	59	51·8	10	20·4	43	31·4	35	27·8	**< 0·001**
Below the EAR (< 70 mg)	280	65·6	55	48·2	39	79·6	94	68·6	91	72·2	
Above UL (> 2000 mg)	0	0·0	0	0·0	0	0·0	0	0·0	0	0·0	**–**
Below the UL (≤ 2000 mg)	427	100·0	114	100·0	49	100·0	137	100·0	126	100·0	
Vitamin D											
Above the AI (≥ 5 µg)	135	31·6	48	42·1	12	24·5	46	33·6	29	23·0	**0·018**
Below the AI (< 5 µg)	292	68·4	66	57·9	37	75·5	91	66·4	97	77·0	
Above UL (> 50 mg)	0	0·0	0	0·0	0	0·0	0	0·0	0	0·0	**–**
Below the UL (≤ 50 mg)	427	100·0	114	100·0	49	100·0	137	100·0	126	100·0	
Vitamin E											
Above the EAR (≥ 12 mg)	175	41·0	53	46·5	17	34·7	58	42·3	47	37·3	0·443
Below the EAR (< 12 mg)	252	59·0	61	53·5	32	65·3	79	57·7	79	62·7	
Above UL (> 1000 mg)	0	0·0	0	0·0	0	0·0	0	0·0	0	0·0	**–**
Below the UL (≤ 1000 mg)	427	100·0	114	100·0	49	100·0	137	100·0	126	100·0	
Vitamin K											
Above the AI (≥ 90 µg)	163	38·2	45	39·5	15	30·6	55	40·1	47	37·3	0·532
Below the AI (< 90 µg)	264	61·8	69	60·5	34	69·4	82	59·9	79	62·7	

AI, adequate intake; EAR, estimated average requirement; UL, tolerable upper limit.

* Kruskal–Wallis test.

† Pearson *χ* ^2^ test.

Post hoc test using Bonferroni correction (a: *P* = 0.05; b: *P* = 0.016; c: *P* = 0.001; d: *P* = 0.011; e: *P* = 0.001; f: *P* < 0.001; g: *P* = 0.002).

Additionally, a significant majority (65·6 %) of the pregnant women had vitamin C intake below the EAR of 70 mg/d. In the total group, the daily median (Q1, Q3) intake of vitamin C was 51·44 (27·74, 87·07) mg, with the mildly (42·40 (25·39, 62·77) mg, *P* = 0·011), moderately (40·88 (24·83, 86·05) mg, *P* = 0·001) and severely food insecure (44·62 (22·85, 75·44) mg, *P* < 0·001) having a significantly lower intake compared with the food-secure participants (71·96 (38·94, 118·18) mg. Similarly, the daily intake of vitamin D was below the AI of 5 µg (200 IU) in 68·4 % of the participants. The median (Q1, Q3) daily intake of vitamin D was 3·61 (1·67, 5·53) µg in the entire group; however, participants experiencing severe food insecurity had a significantly lower intake of 2·49 (1·16, 4·87) µg compared with the food-secure participants (4·49 (2·49, 6·13) µg, *P* = 0·002. Notably, even among the food-secure participants, 59·7 % had intake of vitamin D below the AI, while this proportion was higher (i.e. 72 %) among the food-insecure participants. However, a notably greater proportion of participants had daily intakes of vitamin A (81·0 %), thiamin (86·7 %), riboflavin (74·9 %), niacin (80·3 %), vitamin B_6_ (91·8 %), biotin (75·6 %) and B_12_ (72·1 %) above the EAR and AI.

### Dietary and supplemental intake of minerals

**Figure 2.**
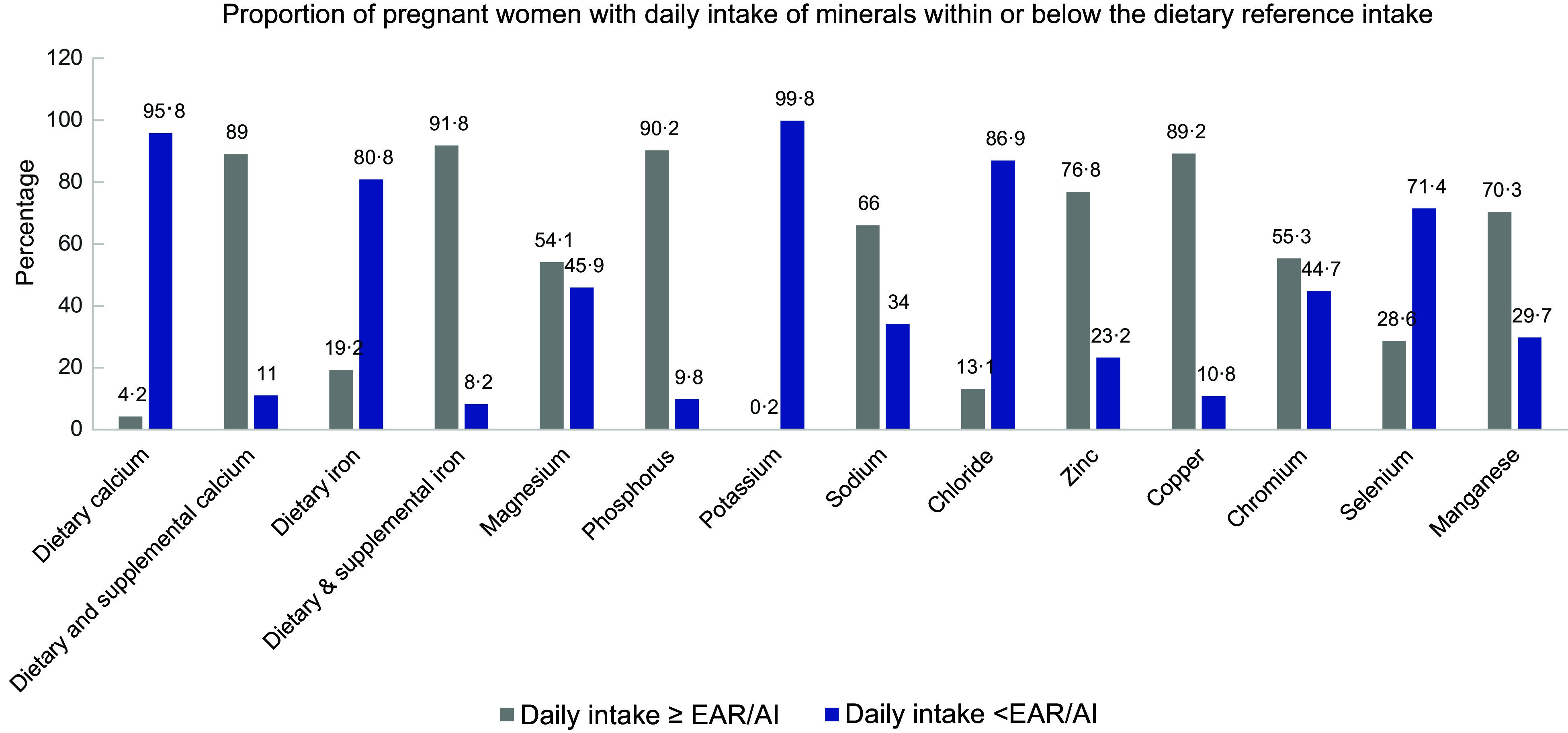
Proportion of pregnant women with dietary and supplemental mineral intakes above or below the daily requirements.

Table 4 and Figure 2 illustrates the intake of selected minerals among the pregnant women described in accordance with their household food security status and dietary reference intakes. In the total group, the daily median (Q1, Q3) intake of dietary Ca was 473·96 (304·47, 648·94) mg, with participants in the food-insecure households having significantly lower intakes than their food-secure counterparts (*P* = 0·001). Additionally, 95·8 % of participants had lower intakes than the daily AI requirement for Ca. However, with the addition of prenatal Ca supplements, the daily intake increased to 1402 (1242–1603) mg, and 89 % met the daily AI requirement of 1000 mg. The daily intake of Fe from both diet and supplements did not differ between the food-secure and insecure participants (*P* = 0·453). Moreover, 80·8 % of the participants did not meet the EAR for Fe from their diet alone. However, this proportion reduced to 8·2 % with the supplementation of Fe. Additionally, the median (Q1, Q3) daily Na intake was 1836 (1255–2748) mg, exceeding the daily AI requirement of 1500 mg. Approximately 66 % of the participants had Na intakes at or above the AI, while 35·4 % had a consumption above the UL of 2300 mg. Furthermore, the intake of chloride was below the daily AI of 2300 mg in 86·9 % of the participants and the median intake varied significantly among the food-secure and insecure participants (*P* = 0·025), as a higher intake was observed among the food-secure than the severely food-insecure participants (1294 (843·08, 1765) mg *v*. 965·70 (526·63, 1555) mg, *P* = 0·029). However, in a greater proportion of the participants (76·8 %), the intake of Zn was above the EAR of 9·5 mg with no difference in the intake between the food-secure and insecure participants (*P* = 0·876).

**Table 4.** Daily dietary and supplemental intake of minerals among the study participants according to household food security status

	Total (*n* 427)		Food secure (*n* 114)		Mildly food insecure (*n* 49)		Moderately food insecure (*n* 137)		Severely food insecure (*n* 126)		
Nutrient per day	Median	Q1, Q3	Median	Q1, Q3	Median	Q1, Q3	Median	Q1, Q3	Median	Q1, Q3	*P* value^*^
Dietary Ca (mg)	473·96	304·47–648·94	560·37^a,b^	369·86, 712·80	455·60	332·74, 671·11	442·42^a^	294·84, 616·17	406·73^b^	250·11, 618·87	**0·001**
Dietary Ca with supplements (mg)	1402	1242–1603	1501	1271, 1653	1398	1281, 1597	1412	1244, 1594	1371	1193, 1548	0·132
Dietary Fe (mg)	16·79	12·67–20·49	16·72	12·18, 20·85	15·86	11·98, 19·87	16·84	13·27, 20·40	16·92	12·55, 21·01	0·891
Dietary Fe with supplements (mg)	80·49	75·46–84·74	79·81	73·99, 84·38	78·86	76·06, 84·17	81·12	77·34, 85·06	80·44	73·45, 84·75	0·453
Mg (mg)	301·28	241·08–377·84	295·73	232·73, 374·56	292·61	226·83, 353·19	295·26	247·40, 376·04	318·33	225·11, 385·08	0·926
Phosphorus (mg)	1020	782·86–1297	1084	841·50, 1382	970·34	876·45, 1217	1010	770·71, 1254·88	949·76	712·32, 1294	0·173
Potassium (mg)	2222	1649–2810	2399	1890, 3101	2165	1731, 2544	2127	1648, 2682	2043	1544, 2890	0·072
Na (mg)	1836	1255–2748	1990	1497, 3017	1800	1291, 2709	2063	1282, 2708	1630	1046, 2681	0·108
Chloride (mg)	1099	675·97 –1742	1294^c^	843·08, 1765	899·76	676·16, 1838	1158	656·36, 1842	965·70^c^	526·63, 1555	**0·025**
Zn (mg)	12·91	9·77–15·97	12·97	9·95, 16·63	12·36	9·16, 14·86	13·27	10·56, 16·00	13·04	9·14, 15·79	0·876
Cu (mg)	1·32	1·03–1·81	1·40	1·11, 1·86	1·25	0·98, 1·61	1·35	1·04, 1·81	1·25	0·94, 1·77	0·425
Cr (µg)	34·16	19·38–54·15	38·96	24·00, 57·81	28·87	21·39, 50·13	30·01	16·66, 58·88	34·60	17·57, 47·89	0·158
Se (µg)	33·33	19·24–52·57	38·68^d^	23·18, 57·72	30·68	19·17, 58·16	33·62	21·21, 52·73	30·03^d^	15·26, 45·03	**0·019**
Mn (µg)	2330	1884–3182	2243	1885, 2937	2277	1758, 3176	2295	1887, 3256	2524	1936, 3412	0·490
Nutrient intake compared with dietary reference intake											
	*n*	%	*n*	%	*n*	%	*n*	%	*n*	%	*P*-value^†^
Dietary Ca											
Above the AI (≥ 1000 mg)	18	4·2	10	8·8	2	4·1	5	3·6	1	0·8	**0·046**
Below the AI (< 1000 mg)	409	95·8	104	91·2	47	95·9	132	96·4	125	99·2	
Dietary Ca with supplements											
Above the AI (≥ 1000 mg)	379	89·0	97	85·1	46	93·9	125	91·2	110	88·0	0·417
Below the AI (< 1000 mg)	47	11·0	17	14·9	3	6·1	12	8·8	15	12·0	
Above UL (> 2500 mg)	1	0·2	1	0·9	0	0·0	0	0·0	0	0·0	0·602
Below the UL (≤ 2500 mg)	425	99·8	113	99·1	49	100·0	137	100·0	125	100·0	
Dietary Fe											
Above the EAR (≥ 22 mg)	82	19·2	22	19·3	7	14·3	25	18·2	28	22·2	0·768
Below the EAR (< 22 mg)	345	80·8	92	80·7	42	85·7	112	81·8	98	77·8	
Dietary Fe with supplements											
Above the EAR (≥ 22 mg)	391	91·8	103	90·4	45	91·8	128	93·4	114	91·2	0·917
Below the EAR (< 22 mg)	35	8·2	11	9·6	4	8·2	9	6·6	11	8·8	
Mg											
Above the EAR (≥ 290 mg)	231	54·1	61	53·5	25	51·0	72	52·6	72	57·1	0·799
Below the EAR (< 290 mg)	196	45·9	53	46·5	24	49·0	65	47·4	54	42·9	
Phosphorus											
Above the EAR (≥ 580 mg)	385	90·2	104	91·2	48	98·0	124	90·5	108	85·7	0·168
Below the EAR (< 580 mg)	42	9·8	10	8·8	1	2·0	13	9·5	18	14·3	
Above UL (> 3500 mg)	0	0·0	0	0·0	0	0·0	0	0·0	0	0·0	–
Below the UL (≤ 3500 mg)	427	100·0	114	100·0	49	100·0	137	100·0	126	100·0	
Potassium											
Above the AI (≥ 4700 mg)	1	0·2	0	0·0	1	2·0	0	0·0	0	0·0	0·102
Below the AI (< 4700 mg)	426	99·8	114	100·0	48	98·0	137	100·0	126	100·0	
Na											
Above the AI (≥ 1500 mg)	282	66·0	85	74·6	33	67·3	95	69·3	68	54·0	**0·011**
Below the AI (< 1500 mg)	145	34·0	29	25·4	16	32·7	42	30·7	58	46·0	
Above UL (> 2300 mg)	151	35·4	48	42·1	15	30·6	51	37·2	37	29·4	0·241
Below the UL (≤ 2300 mg)	276	64·6	66	57·9	34	69·4	86	62·8	89	70·6	
Chloride											
Above the AI (≥ 2300 mg)	56	13·1	16	14·0	6	12·2	21	15·3	13	10·3	0·787
Below the AI (< 2300 mg)	371	86·9	98	86·0	43	87·8	116	84·7	113	89·7	
Above UL (> 3600 mg)	7	1·6	2	1·8	0	0·0	2	1·5	3	2·4	0·861
Below the UL (≤ 3600 mg)	420	98·4	112	98·2	49	100·0	135	98·5	123	97·6	
Zn											
Above the EAR (≥ 9·5 mg)	328	76·8	89	78·1	36	73·5	111	81·0	91	72·2	0·468
Below the EAR (< 9·5 mg)	99	23·2	25	21·9	13	26·5	26	19·0	35	27·8	
Above UL (> 40 mg)	0	0·0	0	0·0	0	0·0	0	0·0	0	0·0	–
Below the UL (≤ 40 mg)	427	100·0	114	100·0	49	100·0	137	100·0	126	100·0	
Cu											
Above the EAR (≥ 0·8 mg)	381	89·2	105	92·1	42	85·7	125	91·2	108	85·7	0·416
Below the EAR (< 0·8 mg)	46	10·8	9	7·9	7	14·3	12	8·8	18	14·3	
Above UL (> 10 mg)	0	0·0	0	0·0	0	0·0	0	0·0	0	0·0	–
Below the UL (≤ 10 mg)	427	100·0	114	100·0	49	100·0	137	100·0	126	100·0	
Cr											
Above the AI (≥ 30 µg)	236	55·3	74	64·9	22	44·9	69	50·4	71	56·3	0·060
Below the AI (< 30 µg)	191	44·7	40	35·1	27	55·1	68	49·6	55	43·7	
Se											
Above the EAR (≥ 49 µg)	122	28·6	41	36·0	16	32·7	41	29·9	24	19·0	**0·048**
Below the EAR (< 49 µg)	305	71·4	73	64·0	33	67·3	96	70·1	102	81·0	
Above UL (> 400 µg)	0	0·0	0	0·0	0	0·0	0	0·0	0	0·0	–
Below the UL (≤ 400 µg)	427	100·0	114	100·0	49	100·0	137	100·0	126	100·0	
Manganese											
Above the AI (≥ 2000 µg)	300	70·3	83	72·8	33	67·3	92	67·2	91	72·2	0·765
Below the AI (< 2000 µg)	127	29·7	31	27·2	16	32·7	45	32·8	35	27·8	
Above UL (> 11 000 µg)	0	0·0	0	0·0	0	0·0	0	0·0	0	0·0	–
Below the UL (≤ 11 000 µg)	427	100·0	114	100·0	49	100·0	137	100·0	126	100·0	

AI, adequate intake; EAR, estimated average requirement; UL, tolerable upper limit.

* Kruskal–Wallis test.

† Pearson *χ* ^2^ test.

Post hoc test using Bonferroni correction (a: *P* = 0.013, b: *P* = 0.001; c: *P* = 0.029; d: *P* = 0.007).

## Discussion

This study aimed to provide a comprehensive description of the energy and nutrient intake of pregnant women in Bloemfontein, compare intakes with the DRI for pregnancy and assess these intakes across household food security categories. In summary, the findings indicate that a greater proportion of the participants had dietary intakes of protein, fibre, vitamins C, D, E and K, pantothenic acid, Ca, Fe, potassium and Se below the EAR, RDA and AI. Nonetheless, the intake of vitamin A, thiamine, riboflavin, niacin, B_6_, B_12_, biotin, Zn and Cu were within the recommended intakes in majority of the participants. Notably, significant differences were observed in the intake of animal protein, total fat, cholesterol, monounsaturated and saturated fat, dietary folate, vitamins B_12_, C and D, chloride as well as Ca across the different household food security categories.

During pregnancy, nutritional and energy requirements increase, particularly during the second trimester, to support fetal development. While energy requirements increase by only 11–15 % compared with pre-pregnancy levels, protein and micronutrient needs rise by as much as 54 %^(24)^. Hence, incorporating nutrient-dense foods and ensuring dietary diversity is crucial to meeting micronutrient requirements without exceeding energy needs^(24)^. In the present study, the median daily energy intake of 8808 KJ was comparable across the various food security groups, indicating that total energy intake is not limited by household food security status among these pregnant women. However, the differences in macro- and micronutrient intake highlight the disparities among the various household food security groups. The energy intake of participants in the present study exceeds the average intake reported among pregnant women in rural KwaZulu-Natal (5773 KJ), which was assessed using two 24-h recalls. Approximately 95 % of the participants in that study fell below their estimated daily energy requirement^(25)^. However, the energy intake in the present study is lower than the energy intake observed in Limpopo, where pregnant women consumed 9406 KJ daily, with most meeting the RDA as assessed from an FFQ^(26)^.

A significant majority of the pregnant women (98·1 %) in our study exceeded the 135 g/d EAR for carbohydrates. This suggests that carbohydrate-rich foods are relatively accessible and affordable, even for food-insecure and low-income households in South Africa. A similar trend was observed in a survey on food purchasing behaviour among South Africans^(27)^. Regarding protein intake, a concerning gap was found. More than half (58·2 %) of the pregnant women consumed less than the RDA of 71 g/d, with lower intakes among the food-insecure households. Furthermore, animal protein intake was significantly lower in the moderately and severely food-insecure groups, reinforcing the challenges of affording nutrient-dense animal-source foods in these households, as a significant majority had a monthly household income below R3000 (approximately $168). Compared with our findings, significantly higher protein inadequacy has been reported among pregnant women in rural KwaZulu-Natal (mean daily intake: 50·4 g, with 86 % below the RDA)^(25)^ and Limpopo, where the average daily protein intake was 30·2 g, notably lower than the 64·81 g observed in our study^(26)^. Beyond economic constraints that may limit access to nutrient-dense protein foods, food choices among pregnant women in South Africa and other parts of sub-Saharan Africa are also influenced by cultural beliefs. Some of these beliefs discourage the consumption of nutrient-rich foods such as meat, eggs, fish and beans due to concerns about their perceived effects on pregnancy outcomes^(28,29)^. Adequate protein intake in pregnancy is essential for the healthy growth and development of the fetus, as well as for the growth of maternal tissues and fetal-support structures like the placenta and extra-embryonic membranes^(30)^. Therefore, optimal protein intake is crucial during this period to prevent poor birth outcomes such as intrauterine growth restriction and low birth weight^(31)^.

In the present study, food-secure participants had significantly higher intakes of total fats, saturated fats, monounsaturated fats and cholesterol compared with their severely food-insecure counterparts. These findings reflect a higher consumption of fat-rich foods, potentially linked to higher access to diverse diets among the food-secure group. Overall, approximately 61 % of participants had daily fibre intake below the AI levels. This observation highlights a broader dietary imbalance, likely driven by insufficient consumption of fibre-rich foods such as fruits, vegetables and whole grains. A report of the diet quality of pregnant women in the NuEMI study indicated a low intake of fibre-rich foods, particularly whole fruits, wholegrains and legumes^(32)^. Similarly, low fruit and vegetable consumption below half of the WHO recommended 400 g per day has been observed among pregnant women in Cape Town and Kwazulu-Natal^(25,33,34)^.

Regarding the intake of vitamins, a greater proportion of participants (i.e. 72·1–91·8 %) had a daily consumption of vitamin A, thiamin, niacin, riboflavin, B_6_, biotin and B_12_ above the EAR and AI. This observation could be attributed to the impact of the Department of Health’s Food Fortification Program, initiated in 2004, that mandates the fortification of maize meal and wheat flour, which are the most common staples consumed in South Africa, with vitamin A, thiamine, riboflavin, niacin, pyridoxine, folic acid, Zn and Fe^(35)^. As observed in this study, a substantial proportion (70 %) of the participants had dietary folate intake below the EAR of 520 mg, with a notably higher percentage among food-secure participants (80 %) compared with their food-insecure counterparts (66 %). Previous studies have similarly reported inadequate dietary folate intake among pregnant women and women of reproductive age in South Africa^(25,33,36)^ and Ghana^(37)^. In South Africa, inadequate dietary folate intake was observed in 74 % of pregnant women in KwaZulu-Natal^(25)^, 98 % of female caregivers in Gauteng^(36)^ and 97 % of pregnant women with gestational diabetes in Cape Town^(33)^. In Ghana, 86 % of pregnant women in their third trimester in Accra, were reported to have dietary folate intake below the daily requirement^(37)^. Inadequate dietary folate intake is prevalent among women of reproductive age in sub-Saharan Africa, increasing the risk of neural tube defects during pregnancy^(7)^. This makes the mandatory supplementation of 5 mg of folic acid as part of antenatal care in South Africa very crucial^(38)^.

A description of the diet quality of our study participants previously reported by Robb *et al.* highlights a pattern of low intake of whole fruits, wholegrain, fish and legumes, and this is reflected in the limited daily intake of vitamin C, D, E and K observed in the present study^(32)^. Specifically, 73 %, 68 %, 72 % and 62 % of the participants did not meet the daily recommended intake for vitamin C, D, E and K, with the food-insecure participants having significantly lower intakes of vitamins C and D. Preconception vitamin C deficiency may be associated with increased risk of premature rupture of membranes (RR:2·2, 95 % CI: 1·1, 4·5, *P* = 0·03)^(39)^, while vitamin D deficiency in pregnancy has been associated with neonatal hypocalcaemia, poor postnatal growth, fragile bones and increased incidence of autoimmune diseases^(40)^. Also, vitamin E deficiency in pregnant women can result in increased free radical production, heightened lipid peroxidation, vascular endothelial damage and increasing the risk of hypertensive disorders^(41)^.

Findings of this study also highlight significantly low dietary Ca and Fe intake among the pregnant women, particularly among those from food-insecure households. The median daily Ca intake of 473·96 mg observed in the present study is considerably lower than the recommended AI of 1000 mg for pregnant women, with 96 % of participants’ Ca intake below the AI. Similarly, 89 % of participants failed to meet the EAR of 22 mg/d for Fe. This trend aligns with findings of previous research conducted in South Africa, where low dietary Ca and Fe intake were reported in over 70 % of pregnant women in both rural and urban settings^(6,25,33)^. A study in Nigeria has similarly indicated suboptimal dietary Ca and Fe intake during pregnancy^(42)^, which is concerning, given the vital role of Ca and Fe in fetal skeletal and brain development and prevention of preeclampsia^(43,44)^. In the South African public healthcare system, supplementation of 1000 mg of Ca and 200 mg of ferrous sulfate (60 mg of elemental Fe) daily is provided to pregnant women as part of routine antenatal care^(38)^.

Another important nutrient necessary for maintaining water and electrolyte balance is potassium. Hypokalaemia in pregnancy resulting from low dietary potassium intake, hemodilution or excessive vomiting can lead to muscle weakness^(45)^. Therefore, AI in pregnancy is essential. As observed in the present study, 99·8 % of the participants did not meet the daily AI for protassium, which is similar to the 98·3 % inadquate intake reported among pregnant women in Cape Town^(33)^. On the contrary, majority of the participants (66·0 %) had Na intake above the AI of 1500 mg, with 35·4 % exceeding the tolerable upper limit of 2300 mg. In a similar study conducted among pregnant women in Johannesburg between 2016 and 2017, 82 % of the participants had daily Na intake (excluding discretionary salt intake) above 2400 mg^(6)^. Generally, South Africans have a high Na intake and a significant burden of hypertension^(46)^. To address this, the South African Government legislated the mandatory salt reduction programme in 2016, which aligns with WHO’s 30 % sodium intake reduction target by 2025^(47,48)^. Data from 2019 show a 1·15 g/d reduction in the intake of salt among South Africans after the salt legislation^(49)^. Although there is not enough evidence associating sodium reduction in pregnancy to prevention of pregnancy-related hypertension and pre-eclampsia, it may be beneficial for women with chronic hypertension or multiple risk factors^(50)^.

This study has some limitations. All dietary intake information was self-reported and relied on participants’ memory, making it subject to recall bias. Moreover, the use of the QFFQ may overestimate or underestimate nutrient consumption. Additionally, per the cross-sectional design of the study, data were collected at only one time point during pregnancy. Given the dynamic nature of dietary habits throughout pregnancy, this design could not fully capture variations in dietary intake across different trimesters. Also, the cross-sectional design of the study limits our ability to infer a causal relationship between household food-security status and nutrient intake. Finally, given that the study was conducted in a tertiary hospital that primarily manages high-risk pregnancies, the findings may not be entirely generalisable to the broader population of pregnant women and should therefore be interpreted accordingly.

In conclusion, this study reveals the widespread shortfalls in the dietary intake of key nutrients, including protein, fibre, vitamins C, D, E and K, as well as potassium among pregnant women in Bloemfontein. The findings also highlight the important role of antenatal Ca, elemental Fe and folic acid supplements in bridging the dietary shortfalls in Ca, Fe and folate intake. However, the study also indicates that most participants met the recommended intake for vitamins A, B_6_, B_12_, thiamin, niacin and Zn. While total energy intake appeared similar across the different food-security categories, significantly lower intakes of animal protein, total fat, saturated and monounsaturated fats, cholesterol, vitamins B_12_, C and D among the food-insecure participants emphasise the influence of food insecurity on nutrient intake. Given the critical role of these nutrients in maternal and fetal health, dietary interventions including nutrition education, food fortification and supplementation, as well as social interventions to alleviate poverty remain crucial strategies for improving the nutritional outcomes of pregnant women.
